# The Collaborative Spark That Ignited the Field of Stromal Stem Cell Biology

**DOI:** 10.3390/bioengineering11070652

**Published:** 2024-06-26

**Authors:** James T. Triffitt

**Affiliations:** Botnar Research Centre, Nuffield Department of Orthopaedics, Rheumatology and Muculoskeletal Sciences, University of Oxford, Nuffield Orthopaedic Centre, Oxford OX3 7LD, UK; james.triffitt@ndorms.ox.ac.uk

Russia has produced many scientists of great renown in a multitude of fields from chemistry, physics, astronautics, and mathematics to biology, pathology, and medicine. In the field of the biology and origins of bone cells, the name of Alexander Friedenstein is synonymous with cardinal concepts of the presence and origins of osteogenic stem cells—the normally quiescent cells that are stimulated to differentiate and generate bone tissue postnatally.

Alexander Friedenstein was born on 24 June 1924 in Kiev, Ukraine [1], just seven years after the Bolsheviks seized power in Russia following the October Revolution of 1917. When he was four years old, his family moved to Moscow where he lived until his death in 1998. This move to Moscow coincided with the death in 1928 of the renowned Russian anatomist and histologist, Alexander Maximov, who was to become Friedenstein’s inspiration in his research activities later in life. Maximov is credited with the formulation of the unitarian theory of blood cell formation whereby the haemopoietic stem cell can develop into all types of blood cells, including myeloid lineage and lymphoid lineage cells. Furthermore, he presented evidence of the obligatory interactions between marrow stroma and haemopoietic stem cells that led to this generation of all blood cells. Maximov noted at the time that stromal cells were associated with an essential haemopoietic-supporting microenvironment that included macrophages, endothelial cells, and reticular cells (fibroblasts). However, he assumed that the effective stromal cells for the provision of this environment were the fibroblasts.

As in all scientific endeavours, most new concepts essentially rely on the past work of others and it is often noted that relatively few ideas presented are unique, but many have been considered by earlier investigators. In Friedenstein’s case, he graciously acknowledged this fact and drew great scientific vision from the earlier work of Maximov [2]. Maximov came from a wealthy family and was considered an aristocrat. Thus, he was increasingly ostracised by the communist regime, which had taken over Russia and assassinated Tsar Nicolas II and his family following the October Revolution. A passionately committed scientist, Maximov eventually made a dramatic escape to the USA to continue his life’s work as an exceptional histologist and scientist in Chicago. A gripping account of this escape and subsequent life in the USA for personal viewing is given in a video on YouTube [3]. 

The Soviet historical mission of discrediting scientists considered to be of the rich, controlling bourgeoisie would likely have been known to Alexander Friedenstein. However, he was working, especially in his later years, in Moscow in slightly more accepting times and in an environment with the cold war between Russia and the West eventually beginning to thaw. Friedenstein noted, in carefully worded terms, in a paper of tribute to Maximov’s ideas on stromal–haematopoietic interrelationships, “… though Maximov was highly respected in the scientific community his concept……was met with particular scepticism” [2].

In the 1960s, the work and support for bone research in particular were very limited in the UK and internationally compared to the current widespread support for work on all aspects of the musculoskeletal system. Hence, few people worldwide were involved with experimental studies on the specific cellular origins of this tissue. The special connection between Friedenstein and Oxford began when Friedenstein’s extensive research became known to Maureen Owen in the MRC Bone Research Laboratory at the Nuffield Orthopaedic Centre in Oxford [4]. He had performed groundbreaking, pioneering work on osteogenic cell differentiation in the 1960s and 1970s [5,6,7,8], together with research on the phenomenon of bone induction by the transitional epithelium [6,9,10,11,12,13,14,15]. 

In May 1977, the main research programme in the MRC laboratory in Oxford was the isolation and functional characterisation of the non-collagenous components of the bone matrix. The laboratory had been newly created from the old hospital laundry at the Nuffield Orthopaedic Centre and comprised just five individuals at that time: Maureen Owen and James Triffitt as MRC Scientific Staff with one technician each and a part-time group histologist. In October 1977, under the leadership of Maureen Owen, we also started a new line of research on the differentiation of osteogenic tissue, which became a major interest. Some work on this subject had been performed in the MRC Bone Research laboratory, previously named the Bone-Seeking Isotopes Research Unit at the Churchill Hospital, Oxford, for several years up to 1972. Owen’s previous work [16] on the origins of osteoblasts in bone formation was initiated following her accompanying of her husband on sabbatical leave, in 1962–1963, from Oxford to the Brookhaven National Laboratory in Long Island, New York. Whilst there, she learned of the work of Sherman and Quastler, experts in cell and tissue kinetics who used tritium-labelled thymidine to trace proliferating cells by using autoradiography [17]. These procedures were being used by Tonna and Cronkite in the Brookhaven Laboratory to study cell proliferation in the femoral periosteum in mice of different ages, before and after fractures [18]. Owen’s first venture upon returning to Oxford was to study the cell population kinetics and histogenesis of rapidly growing bone in vivo in much greater detail. This was achieved by using very young rabbits, aged 2 weeks, by using similar radioactively labelled thymidine and glycine moieties with detection by autoradiographic techniques [19,20]. These were classical studies using this precise technology on histological sections that demonstrated in great detail the rapid rate of bone formation in these animals, the mode of histogenesis, and the exact location and progression of differentiation in vivo of the most primitive cells of bone. Thirty years later, recognition of the significance of her careful and tedious studies on the different functional states of bone cells was justly recognised by being reprinted as a classic article in a prestigious orthopaedic journal [21].

A new approach to the problem of studying osteogenic differentiation was afforded and stimulated by the pioneering work of Friedenstein and his colleagues [4]. They had shown that bone marrow cells plated out in in vitro culture give rise to fibroblastic colonies, with each colony shown to be derived from a single fibroblastic colony-forming cell, the “FCFC” (Figure 1a). Either marrow cells or fibroblasts cultured from marrow tissue, when implanted in a diffusion chamber in vivo, were shown to differentiate to form a calcified tissue which resembles bone. The evidence suggested, therefore, that the stromal fibroblasts of marrow contain the osteogenic stem cells and their progeny. We began to use this system in the laboratory to investigate the factors which stimulate and regulate differentiation of the osteogenic cell line and to study the biosynthesis of calcified tissue in normal and abnormal situations in both human and experimental animals. 

The general aims of work in Oxford at the time (mid-1980s) at the MRC Bone Research Laboratory was to obtain more information about the processes of osteogenic cell differentiation and the biosynthesis and calcification of skeletal tissue, with the ultimate purpose being to increase understanding of the normal situation and of the conditions operating in skeletal repair and in diseases arising from disturbances in bone cell function. A number of other groups in the UK were interested in the study of marrow stromal cells, but mostly from the haematology standpoint as a major part of the supportive haemopoietic stem cell microenvironment [22,23,24,25]. However, at this time, the specific objectives in the Oxford laboratory were mostly related to the work of Friedenstein. In particular, they were (i) to confirm the seminal work of Friedenstein and to provide a reproducible assay for osteogenic stem cells, (ii) to determine the distribution of the stromal stem cells within the bone marrow, (iii) to determine the effect of calcium-regulating hormones on osteogenic stem cell differentiation, (iv) to develop the diffusion chamber system for the study of osteogenic cell differentiation and bone formation by marrow cells from normal human subjects and from individuals with certain bone disorders, and (v) to study the matrix elaborated by the osteogenic cells in diffusion chambers with time after implantation, with special reference to the known bone-specific proteins. 

It was with this research plan in mind that a letter of invitation was sent by Maureen Owen to Alexander Friedenstein to visit the Oxford laboratory for research discussions. On a number of occasions over the years, he attempted to get permission from the Russian authorities to travel, but as he was Jewish, he was always under pressure and his requests were rejected. Jews had been, through history from the 15th century, subjected to state policies of isolation and control in Russia. It is believed that on one occasion, even when he got the permission to travel, he was briskly removed from the plane by the authorities just before departure. However, towards the end of the 1980s under the influence of Gor-bachev’s leadership of the USSR, the controls on permitted travel became less restrictive. Hence, eventually in around 1991, despite the turmoil resulting from the imminent collapse of the USSR and Gorbachev’s resignation with the later replacement by Yeltsin as President of the new Russian Federation, Alexander Friedenstein was able to arrive in Oxford for a few day’s visit. He greatly enjoyed staying in the home of Drs. John and Maureen Owen, which was located in the central historic part of the city. This was for a short visit of a couple of days and he visited the laboratory briefly only once, meeting the laboratory staff and technicians under the close supervision of Maureen Owen. Our laboratory had grown in numbers of postdoctoral researchers, international visitors, and DPhil students by this time and a wide variety of topics were openly and intensely discussed. It was an interesting and stimulating experience for all involved, including Alexander Friendenstein, with ideas on bone induction and osteogenesis being central (Figure 1b). Discussions ranged from using the stromal stem cells in their capacity for haematopoietic support, and hence to their production of cellular factors, to the generation of skeletal tissues for correction of the many orthopaedic problems caused by disease or traumatic conditions. 

A memorable discussion concerned the series of critical experiments for his theory and proof of the concept that osteogenic stem cells existed [26]. These were meticulous and time-consuming experiments using young adult rabbit marrow over an appreciable time frame and, to my knowledge, these have never been reproduced in their entirety. Here, he isolated clones of marrow fibroblasts and implanted them in vivo in semipermeable, but completely cell-impermeable, chambers as originally formulated by Algire [27]. Not only were individual clones used, but also amplified populations from sequentially passaged fibroblasts. This showed that cells within these populations had extensive proliferative activity and most importantly retained their osteogenic activity with the essential characteristics of stem cells [26].

Every possibility for the future use of these cells for therapy was considered from the obvious potentials for orthopaedic reconstructions to the uses for penile implants for treatment of erectile disfunction. He conveyed a wide appreciation of a multitude of potential benefits and the enthusiasm and excitement of continuing intense future research on these primitive stromal cells.

However, most of his time in Oxford was spent away from the laboratory with Maureen Owen discussing concepts related to stromal cell differentiation and comparisons of UK and Russian life. On one occasion, he was particularly taken aback when he learned what, he perceived, was the very large salary of a young junior animal technician who he met in the Nuffield Orthopaedic Hospital facility. It was related that, after he had been left alone with this technician for a short time, he enquired whether he would have been able to earn as much if he came to the UK. With the disintegration of the USSR, those living in Moscow suffered great hardship and poverty and perhaps his questions were understandable. But this emphasised how difficult it must have been in his own country to pursue scientific endeavour and his research work with severely limited resources. Hence, his achievements and creative concepts that he formulated by his profound devotion to his scientific endeavours are all the more remarkable and admirable.

The relatively sparse but close connection and sharing of ideas between the work of Friedenstein and Owen was highly significant and resulted in a number of joint publications [28,29,30]. They were the catalyst that subsequently opened up the Western world view and initiated more intense study of the mechanisms of differentiation and the nature of the primitive cells involved postnatally in the formative and regenerative processes of skeletal tissues, as well as the wider view of a stromal cell system permeating most organs of the body, which could allow or stimulate regenerative processes, especially in the musculoskeletal system. Furthermore, the concepts of the inductive process proposed by the work of Urist at UCLA and his predecessors indicated that almost all organs may harbour cells in the tissues that could become osteoblasts and form skeletal tissues [31].

Friedenstein’s work on both these aspects of committed and inductive potentials of stromal cells and on the application of novel experimental approaches to discovering significant central aspects of bone cell differentiation were reasons for the major initiation of research on these topics in the Oxford laboratory. Furthermore, the close association of Owen’s views and those of Friedenstein and their mutual respect led to an explosion of interest and work in the field. Subsequently, around this time, in 1992–1993, Friedenstein was able to travel to the USA to work for an extended period with Pam Robey’s group at the NIDCR Branch of the National Institutes of Health in Bethesda, Maryland. There, he influenced significant changes in emphasis from study of model osteoblastic cancer cell lines to investigations on normal animal and human cells to unravel skeletal cell biology. Both detailed and more general accounts of the overall historical development of the concept of stromal stem cells are given in a number of earlier publications [32,33,34,35,36].

When Maureen Owen retired in 1993, I was able to organise a meeting of the Bone and Tooth Society at Keble College, Oxford, in her honour, and Friedenstein was able to travel from the USA to accept the invitation to make a second visit to Oxford at the age of 69 years. He attended the meeting along with a number of renowned international experts in the bone field of the time (Figure 2). Alexander was highly regarded for his life’s work by all present and the mutual high esteem between himself and Maureen Owen was evident during this second short visit. It was with great sadness that, just four years later, we learned of his tragic death alone in Moscow in which a high proportion of citizens still lived below subsistence levels.

Alexander Friedenstein’s original thinking, technical expertise, and constant devotion to scientific endeavour during the harsh conditions that persisted during his lifetime are a shining example to all. The legacy of Alexander Friedenstein will remain as a father figure and unique pioneer in the stem cell field of regenerative medicine.

## Figures and Tables

**Figure 1:**
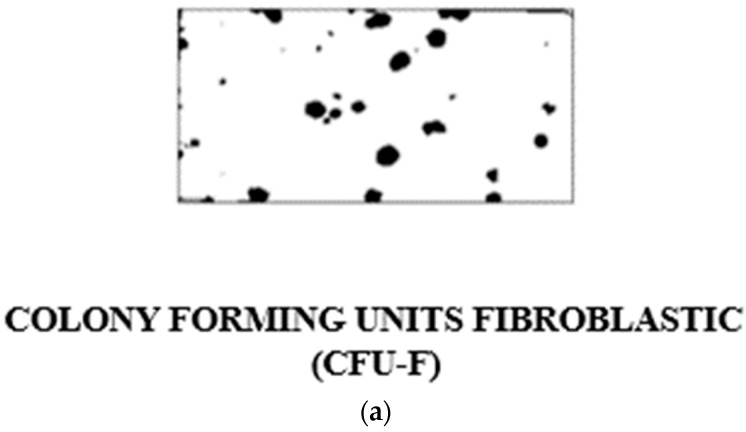

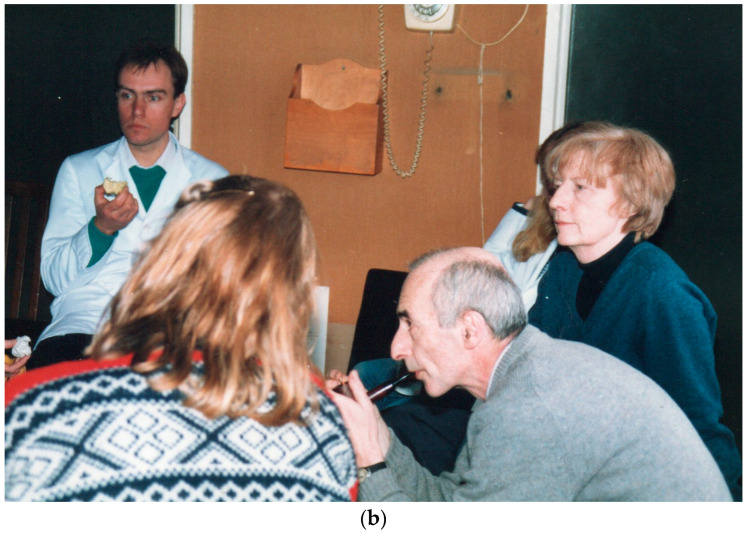
(**a**) Portion of a tissue culture flask showing stained colonies of marrow stromal fibroblasts derived from single-cell suspensions of young rabbit bone marrow growing as individual colonies; the so-called “colony forming units fibroblastic” (CFU-F) or “Fibroblast Colony Forming Cells” (FCFC). (**b**) Alexander Friedenstein in earnest discussion in Oxford in the Nuffield Department of Orthopaedics seminar room on the roof of the main building of the Nuffield Orthopaedic Centre on his visit to the MRC Bone Research Laboratory. This building was demolished when the new hospital was constructed on this site (2002–2005). From left to right: Clive Joyner, Helen Mardon (back view), Alexander Friedenstein, Maureen Owen. Notice Alexander Friedenstein enjoying his pipe. Smoking was allowed in hospitals in England until July 2007.

**Figure 2:**
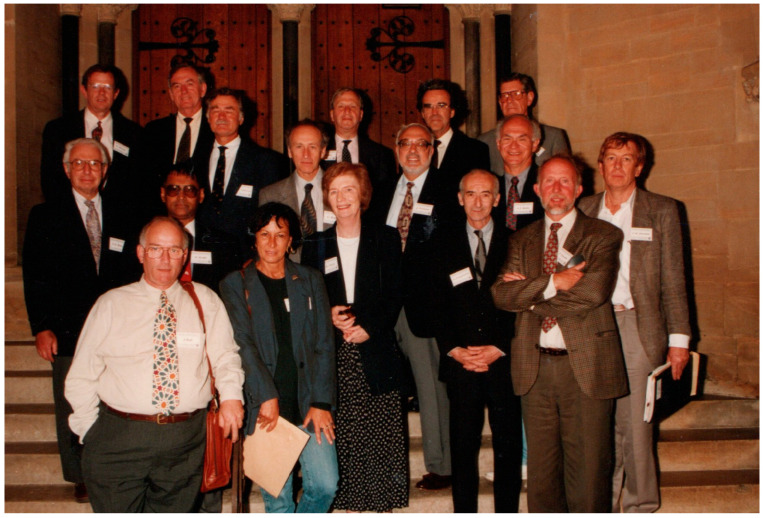
Leading bone experts attending the 1993 July meeting of the Bone and Tooth Society held in honour of Dr. Maureen Owen’s retirement. This meeting was held in the University Museum lecture theatre and Keble College Oxford. The photograph was taken on the entrance steps to the Museum. Front row (from left): Itai Bab (Israel), Alberta Zambonine-Zallone (Italy). Second row (from left): Larry Raisz (USA), Hari Reddi (USA), Maureen Owen (UK), Alexander Friedenstein (Russia), Peter Nijweide (Holland). Third row (from left): Gidean Rodan (USA), John Termine (USA), Arnie Kahn (USA), Rolfe Howlett (Australia). Fourth row (from left): Greg Mundy (USA/Australia), Jack Martin (Australia), Clarke Anderson (USA), Steve Krane (USA), Herbie Fleisch (Switzerland), Gastone Marotti (Italy).
